# Learning-Based Discontinuous Path Following Control for a Biomimetic Underwater Vehicle

**DOI:** 10.34133/research.0299

**Published:** 2024-01-30

**Authors:** Yu Wang, Hongfei Chu, Ruichen Ma, Xuejian Bai, Long Cheng, Shuo Wang, Min Tan

**Affiliations:** ^1^ State Key Laboratory of Multimodal Artificial Intelligence Systems, Institute of Automation, Chinese Academy of Sciences, Beijing, China.; ^2^ School of Artificial Intelligence, University of Chinese Academy of Sciences, Beijing, China.; ^3^ School of Electrical Engineering, Liaoning University of Technology, Jinzhou, China.

## Abstract

This paper addresses a learning-based discontinuous path following control scheme for a biomimetic underwater vehicle (BUV) driven by undulatory fins. Despite the flexibility of the BUV motion, it faces the challenge of dealing with discontinuous paths affected by irregular seafloor topography and underwater vegetation. Therefore, BUV must employ path switching strategy to navigate to the next safe area. We introduce a discontinuous path following control method based on deep reinforcement learning (DRL). This method uses the line of sight (LOS) navigation algorithm to provide the Markov decision process (MDP) state inputs and the soft actor-critic (SAC) algorithm to train the control strategy of the BUV. Unlike the traditional fixed waveform control method, this method encourages the BUV to learn different waveforms and fluctuation frequencies through DRL. At the same time, the BUV has the ability to switch to a new path at necessary moments, such as when encountering underwater rocks. The results of simulations and experiments demonstrate the successful integration of the undulatory fins with the SAC controller, showcasing its efficacy and diversity in discontinuous underwater path following tasks.

## Introduction

Covering 71% of the Earth’s surface, the vast ocean holds a wealth of natural resources that continues to captivate human curiosity. To delve deeper into the mysteries of the ocean, scientists and engineers have tirelessly developed autonomous underwater vehicles (AUVs) capable of independent navigation [1]. Due to the complexity and variety of the underwater environment, underwater motion and operation require excellent maneuverability and disturbance resistance. With millions of years of natural evolution as a testament, aquatic creatures have honed exceptional underwater locomotion abilities, providing a profound source of inspiration for designing high-performance biomimetic underwater vehicles (BUVs). By emulating the motion and morphology of fish and other marine species, researchers unlock new possibilities for AUV design and operation.

In comparison to conventional axial propeller-driven AUVs, BUVs exhibit superior energy efficiency while minimizing disturbances to the marine ecosystem, a critical aspect of preserving marine life. These vehicles achieve these by drawing from the propulsion techniques of fish, where two primary modes stand out: body/caudal fin (BCF) and median/paired fin (MPF) propulsion [2]. Numerous fish species adopt the BCF method, allowing them to attain substantial forward thrust and remarkable speed. An exemplar representative of BCF-driven fish is the dolphin, which has streamlined body and powerful tail fin. Conversely, certain fish rely on MPF propulsion, generating less thrust but boasting enhanced motion stability. MPF propulsion is well suited for navigation in complex underwater environments. The batfish serves as an outstanding example of MPF-driven fish, leveraging its paired fins to gracefully maneuver amidst challenging underwater terrains.

The design of BUV draws inspiration from the diverse swimming techniques of different fish species and various aquatic locomotion mechanisms. The sinusoidal wave motion of the black ghost knifefish’s anal fin generates propulsive thrust, inspiring the concept of undulatory fins for underwater propulsion. This design enables BUVs to exhibit different motion modalities [3]. The application prospects of undulatory fins have garnered widespread interest and research dedication from scientists and engineers. Zhang et al. [4] designed a wave-like fin with a flexible fin surface that approximated a sinusoidal vibration pattern, and its hydrodynamic analysis was conducted. Sfakiotakis et al. [5] developed a wave-like fin device comprising eight interconnected parallel bellows actuators (PBAs) with flexible material interconnections. The PBA allowed for bending motions in any planar direction. Wang et al. [6] also engineered an embedded shape memory alloy (SMA) wire-driven robot pectoral fin, capable of mimicking the motion of muscle fibers for three-dimensional movement. In our laboratory, a series of BUVs with undulatory fins were also designed and built to study in this domain [7–10].

In this paper, our BUV is equipped with two sets of the flexible undulatory fins on both sides. Unlike other undulatory fins [11,12], the flexible undulatory fins features long, thick, and highly elastic fin membranes with a uniquely designed shape that allows for remarkable flexibility during the oscillation process. For path following task, several mature methods exist for underwater autonomous vehicles (AUVs). Traditional control methods [13–15] are widely used for AUVs driven by axial propellers. However, due to the strong coupling forces generated in different directions by undulatory fins, applying these methods to our BUV poses substantial challenges.

In order to effectively control the motion of bionic robots, it is necessary to explore the motion characteristics of them. For example, Li et al. [16] combined the growth adaptability of vine plants with a coordinated control system so that their bionic soft robot can move in a very complex environment. Li et al. [17] created an aerial-wall robotic insect based on the movement of insects landing, climbing, and taking off on vertical surfaces. In this paper, we draw inspiration from deep reinforcement learning (DRL) as it offers a viable approach to train an agent in an interactive environment to learn the control task. Using DRL, we can overcome some limitations of traditional methods and explore more properties of bionic propulsion. We aim to design an end-to-end controller that directly generates control parameters as output based on the current state information after observing environmental cues. Several DRL methods have been proposed for path following control of underwater vehicles. For instance, Wu et al. [18] proposed a technique based on the deterministic policy gradient (DPG) algorithm to train their AUV to achieve depth control by following desired depth trajectories. Ma et al. [19] introduced an actor model critic (AMC) architecture that embeded neural network models into the traditional actor-critic framework. Their experiments demonstrated significantly lower tracking errors of controllers based on this method, regardless of ocean currents. Wang et al. [20] proposed a path following control method based on the simplified deep deterministic policy gradient (S-DDPG) algorithm. S-DDPG considered only the immediate reward of the current state, eliminating the need to predict future rewards, which reduced the generation of irrelevant failure samples and improved controller training speed. While these methods prove effective in simulation scenarios, they also have some limitations. For instance, Wu et al.’s [18] method required setting different Markov decision processes (MDPs) for subsequent tasks, leading to significantly increased computational costs and limited applicability of each control strategy. Although Ma et al. [19] conducted comparative tests with and without water flow interference in a simulation environment, they did not validate the method in a real environment. While Wang et al.’s [20] method achieved the control objectives, there remained a need for further improvement in the stability of the control outputs.

In this paper, a new learning-based discontinuous path following control is successfully achieved on our BUV. The main contributions are summarized as follows:

1. A SAC reinforcement learning algorithm combined with a task switching mechanism is proposed as a control scheme to achieve discontinuous path following on our BUV.

2. Controller perceptrons are designed to imitate neural interactions of fish swimming in the environment, which allow the BUV to explore a variety of different ways of fluctuating.

3. Experiments are conducted in an indoor pool environment. The results reveal the practicability and effectiveness of our control scheme.

In the remainder of this paper, the design of the flexible undulatory fins is introduced in the “Biomimetic Propulsor Description” section. The BUV and the control task are introduced in the “System Description” section. The path following control scheme and the DRL frame are described in the “Control Scheme” section. Experimental results are presented and analyzed in the “Experimental Results” section. In the end, the paper is concluded in the “Conclusions” section.

## Results

### Experimental Results

The path following experiments are conducted in an indoor pool with dimensions of 5 m × 4 m × 1.5 m (length × width × depth). The real-time positions and velocities of the BUV can be obtained by a global visual tracking system, which is also used in [23–25].

**Fig. 17.**
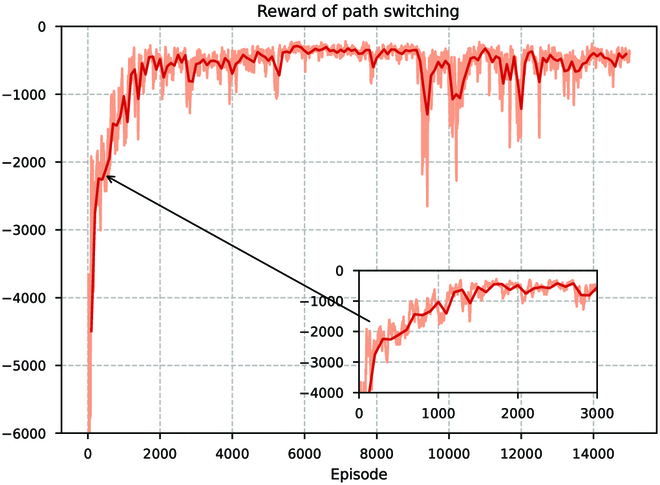
Training curves for cumulative reward values during path switching controller training.

**Fig. 18.**
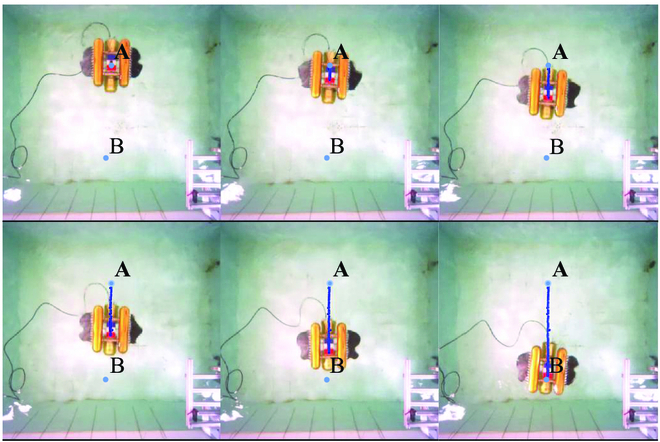
Point to point task of the BUV in path switching experiments.

We first train the path switching controller, and the reward changes during training are shown in Fig. 17. It can be seen that the training is basically reaching convergence after 2,000 rounds of iterations. Since the simulation environment is modeled according to a 1:1 hydrodynamic model, we validate the performance of this controller by applying it to the BUV and designing a path switching task between two points after reaching convergence in the simulation. The experimental results are shown in Fig. 18. The BUV accomplishes the point-to-point path-point target switching under the control of this controller.

**Fig. 19.**
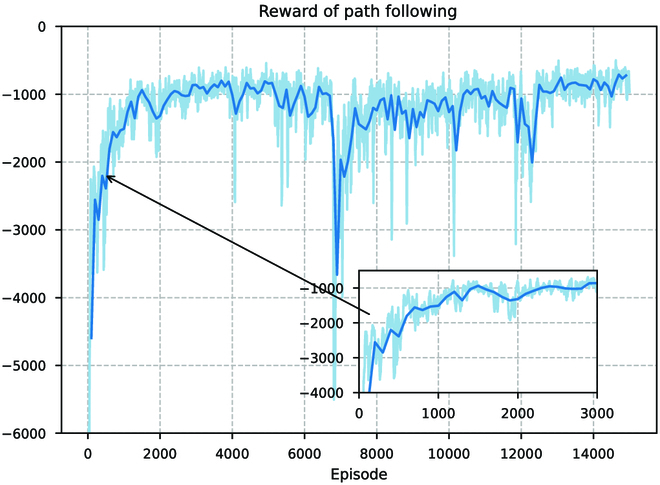
Training curves for cumulative reward values during path following controller training.

**Fig. 20.**
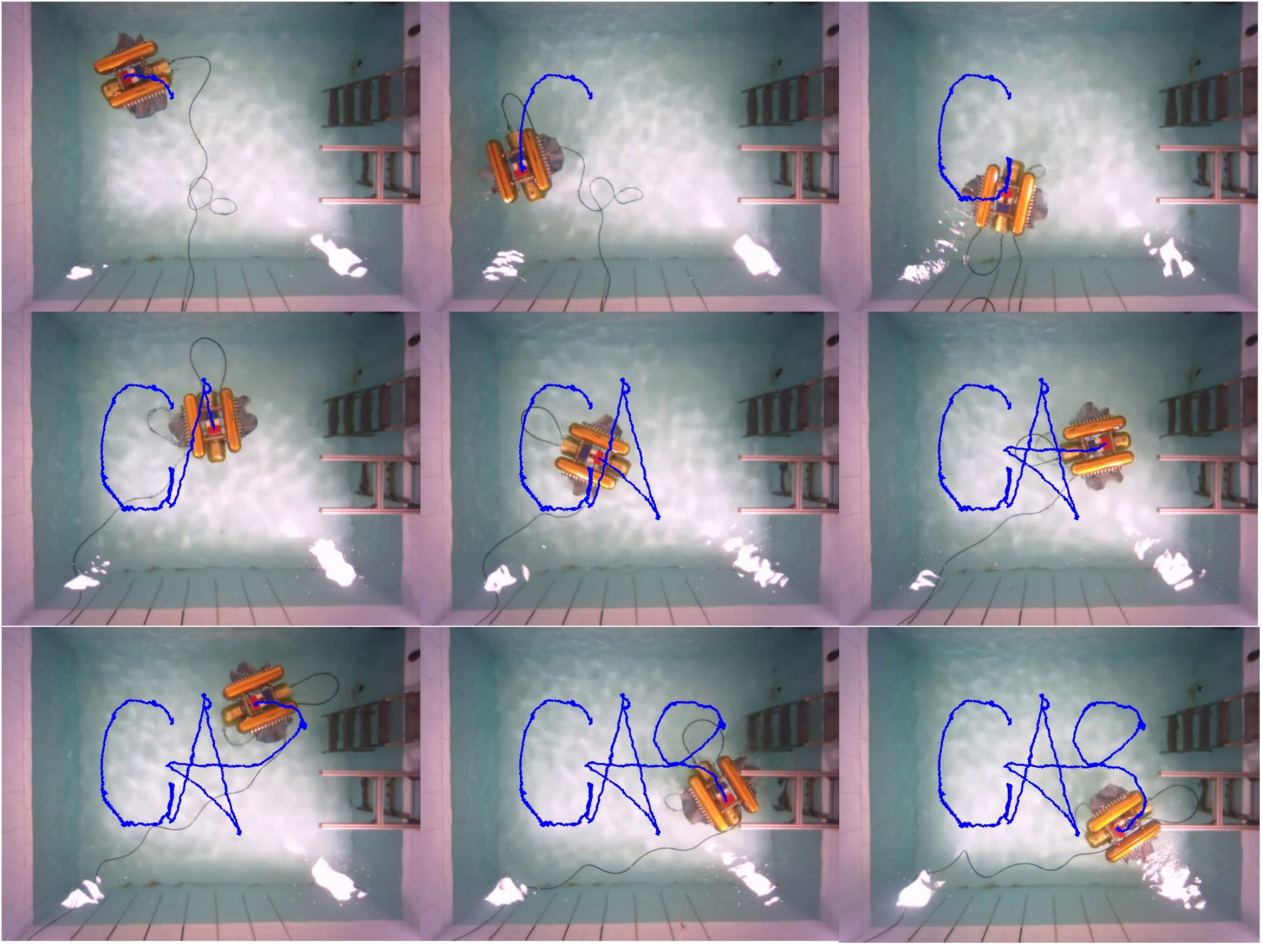
Trajectories of the BUV in path following and path switching experiments.

**Fig. 21.**
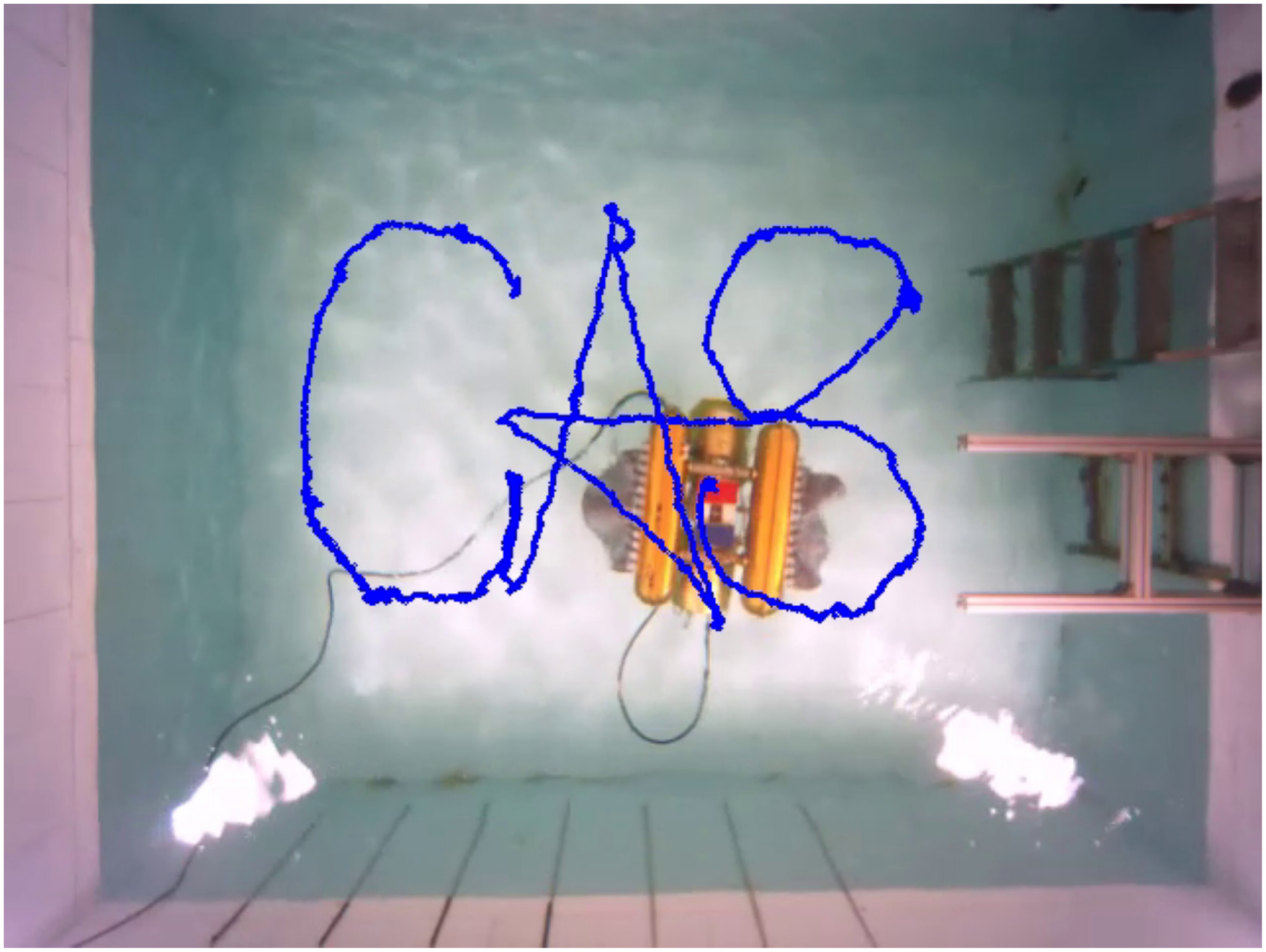
The final tracking trajectory of the BUV.

We then train the path tracking controller, and the results are shown in Fig. 19. Due to the increased difficulty of the control task and the exploration of suboptimal control strategies, although the path following controller reaches stability around 2,000 iterations, it fluctuates more in the later training process. Next, we design the reference paths that are “CAS”-shaped lines approximated by *Be*′*zier* curves to validate the performance of the two controllers. The preferred speed is 0.07 m/s. We deploy the controllers that have been trained to track the reference paths in real-world scenarios. The tracking process for the discontinuous path of the “CAS” shape is shown in Fig. 20, and the final tracking path is shown in Fig. 21.

**Fig. 22.**
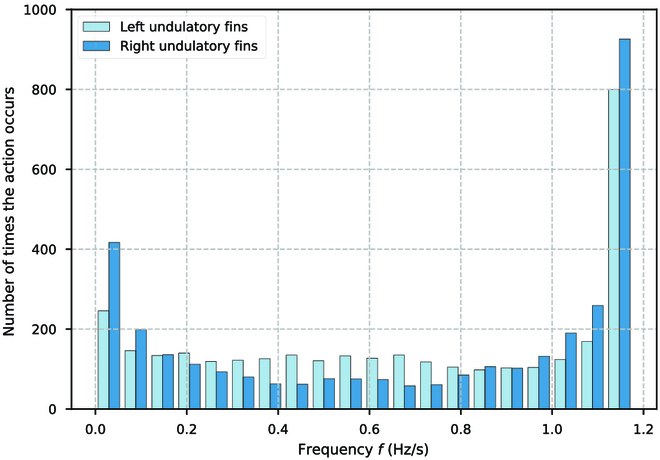
Frequency of fluctuation of the left and right undulatory fins during path tracking.

**Fig. 23.**
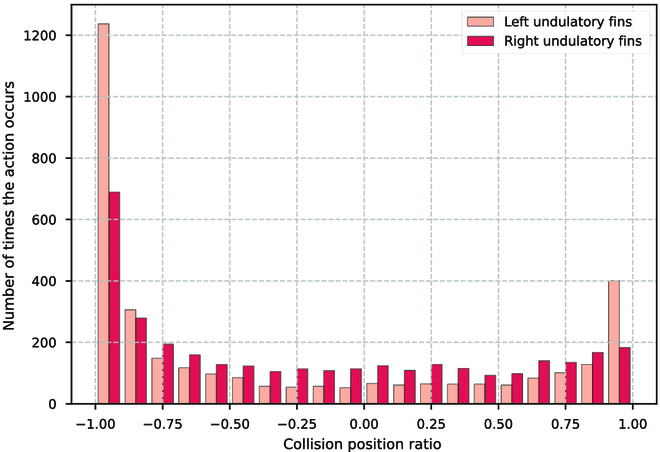
Collision position ratio of the left and right undulatory fins during path following.

During the tracking process, the BUV constantly observes the environment and acquires real-time state information, which is then fed into the path following controller. The controller processes this information through the MLP to determine the appropriate control actions needed for precise path tracking. Similarly, when requiring path switching or specific maneuvers, the BUV utilizes the path switching controller. This controller, with the help of MLP, generates control parameters for seamless transitions between different paths. We collect the amount of action during tracking as shown in Figs. 22 and 23. It can be seen that compared to the set maximum frequency of 1.7 Hz, the control strategy gives a lower fluctuation frequency of up to 1.2 Hz. This is related to the size of the control task, and the tracking accuracy is more tested in the “CAS” tracking task. Moreover, the control strategy prefers forward movement, and backward movement is only considered when fine-tuning. The closer the collision position is to −1 indicates that the traveling wave pushes into the opposite direction of the BUV’s forward direction to a greater extent, giving the BUV more forward propulsion.

**Fig. 24.**
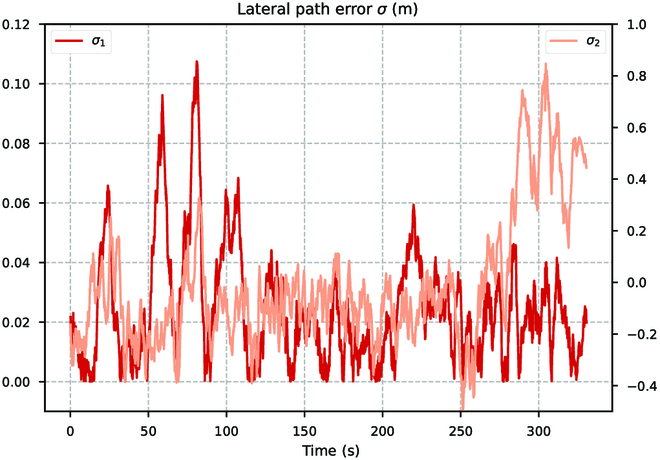
The average lateral path errors in repetitive tests (the same scene).

**Fig. 25.**
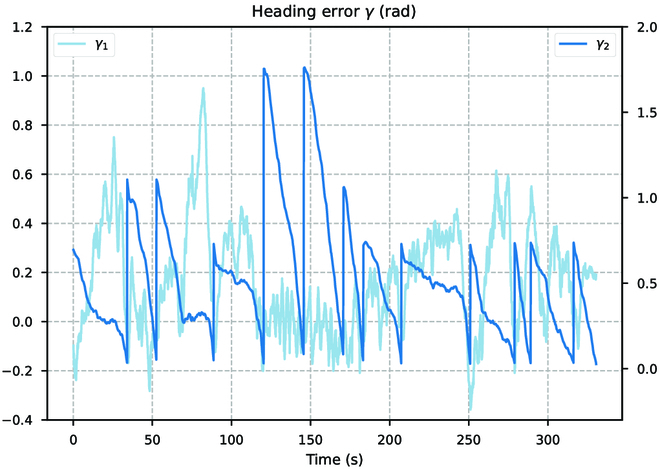
The average heading errors in repetitive tests (the same scene).

As shown in Fig. 24, we calculate the lateral errors *σ*_1_ and *σ*_2_ during the tracking process. It can be observed that the lateral path errors are concentrated within the range of 0 to 0.03 m, which are approximately within 1% of the scale of the tracked trajectory. Where the lateral error fluctuations are large is due to abrupt changes or large curvature in the tracking path. During the tracking process, this reason can also cause the current orientation of the BUV to deviate from the direction of the trajectory. But the BUV can quickly respond and adjust its heading in time using the control strategy learned from reinforcement learning, as shown in Fig. 25. After a sudden change in direction ($\gamma_{2}$), the BUV can quickly change its heading. Considering that the experiments are conducted in real-world environments, some uncertainties and external disturbances may affect the path following performance. Despite these challenges, the performance of the proposed controllers in this study remains remarkably robust and effective.

The low magnitude of lateral path errors and heading errors demonstrates the high precision and accuracy achieved by our control system. The controllers’ ability to maintain the BUV within such a small deviation from the desired paths highlights their exceptional tracking capabilities. In the face of realistic disturbances and variable underwater environments, BUV can perform the task of path tracking very well. These results validate the effectiveness and reliability of the end-to-end control design.

## Methods

### Biomimetic Propulsor Description

This section presents a description to the design of the flexible undulatory fins inspired by the swimming pattern of black ghost knifefish, including its driving system (see the “Driving system design” section) and the undulatory fins (see the “Design of the undulatory fins” section).

#### Black ghost knifefish swimming pattern

**Fig. 1.**
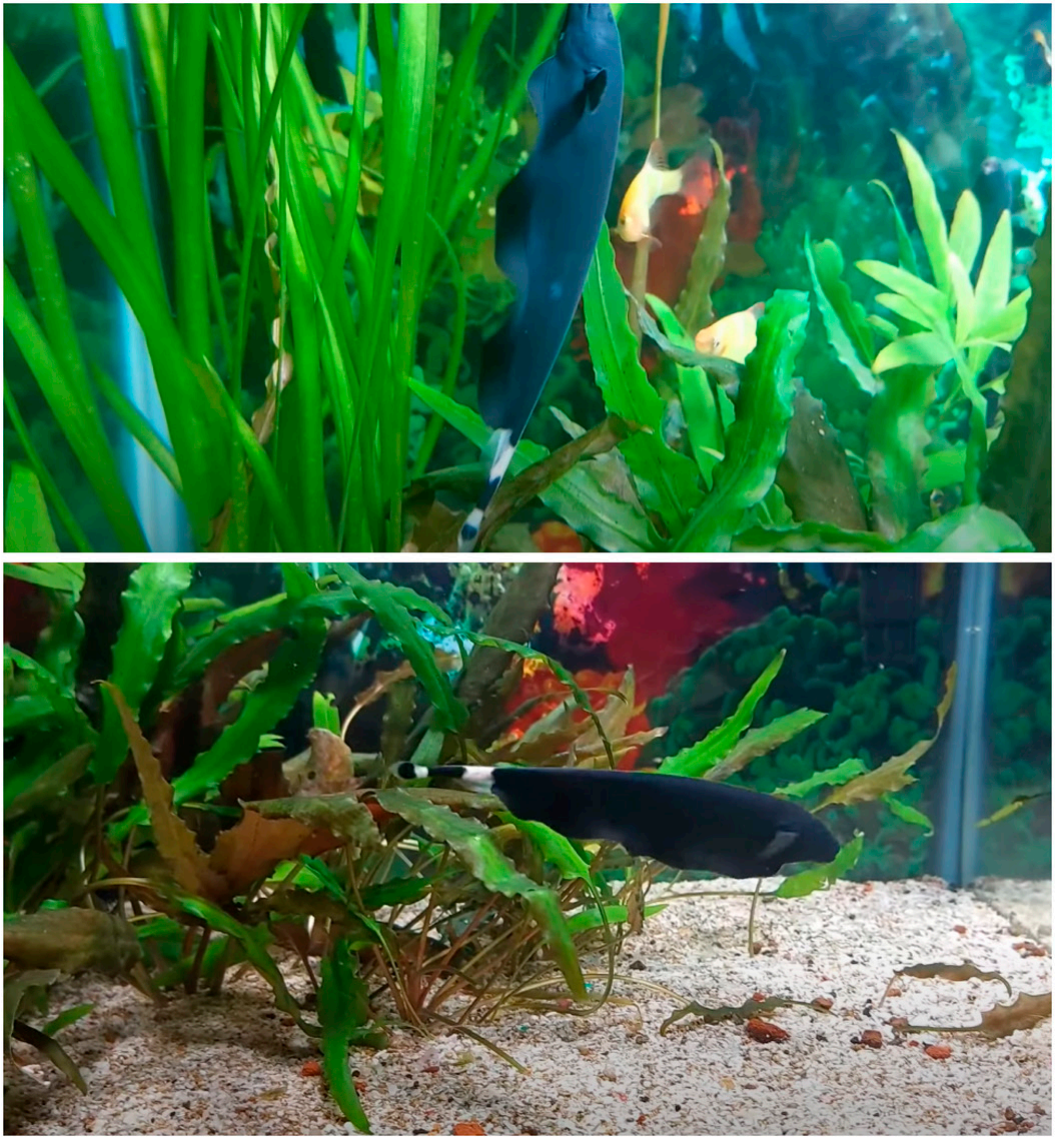
Black ghost knifefish’ swimming motion.

**Fig. 2.**
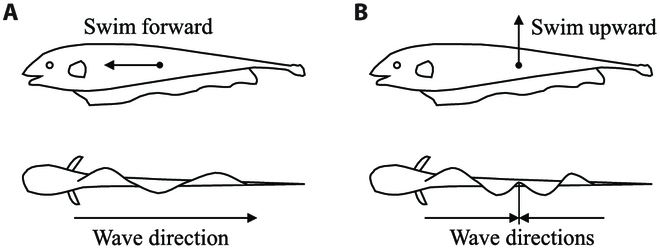
Side and bottom schematics of the black ghost knifefish when it (A) generates a sinusoidal wave to swim forward and (B) generates inward counterpropagating sinusoidal waves to swim upward or hover.

The black ghost knifefish’s ventral flag-like anal fin, characterized by its large and well-developed wave-like structure, enables it to perform impressive backward and vertical movements, as depicted in Fig. 1. For the unique swimming style of the black ghost knifefish, the fluctuating form of the undulatory fins is simplified into two distinct waveforms. As shown in Fig. 2, when the fish needs forward or backward momentum, the undulatory fins show the sinusoidal-like waveforms. The waveforms fluctuate in the forward or backward direction and push the water backward or forward. Thus, the undulatory fins get propulsion in the opposite direction. When the fish needs upward or downward momentum, the undulatory fins’ fluctuating form is similar to the collision of two symmetrical sinusoids. Thus, the undulatory fins generate propulsion in the downward or upward direction.

#### Driving system design

**Fig. 3.**
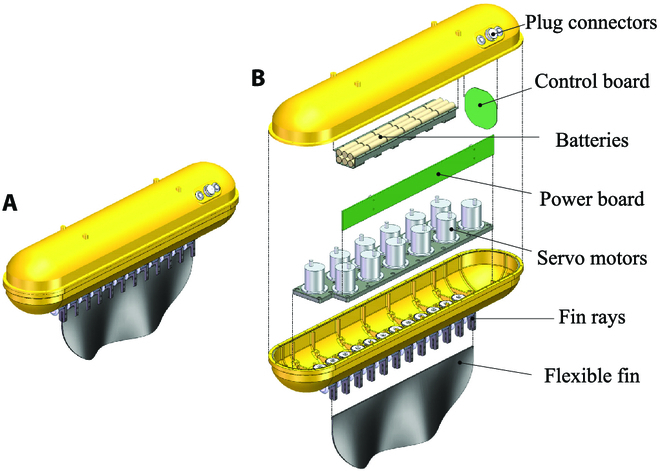
Design of the flexible undulatory fins. (A) External view. (B) Explosive view.

**Fig. 4.**
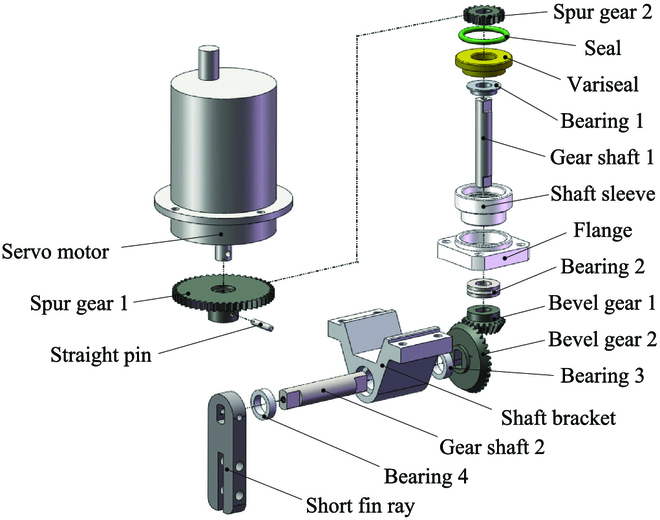
Explosive view of a fin ray module.

To actuate the undulatory fins and generate waves for underwater propulsion like the black ghost knifefish, the flexible undulatory fins has been designed as shown in Fig. 3. The propulsion system is modular designed, has a control system and a power system, and can be independently used as an underwater vehicle. It possesses 12 fin ray modules; the design of the module is shown in Fig. 4. An industrial ratio-controlled servo motor (Udoservo UD-50F) is used to actuate the short fin ray through a two-step gear transmission. The motor has a maximum output torque of 50 kg/cm and a maximum rotation angle of 90^∘^. To increase the rotation angle of the fin ray, the transmission ratio of gears is designed as 9 : 16 so that each fin ray can rotate in a range of 160^∘^, while its output torque can still reach 28.125 kg/cm.

#### Design of the undulatory fins

Multimodel waves can be generated on the undulatory fins by assigning the rotation angles of fin rays to sequential phases of different wave patterns.

**Fig. 5.**
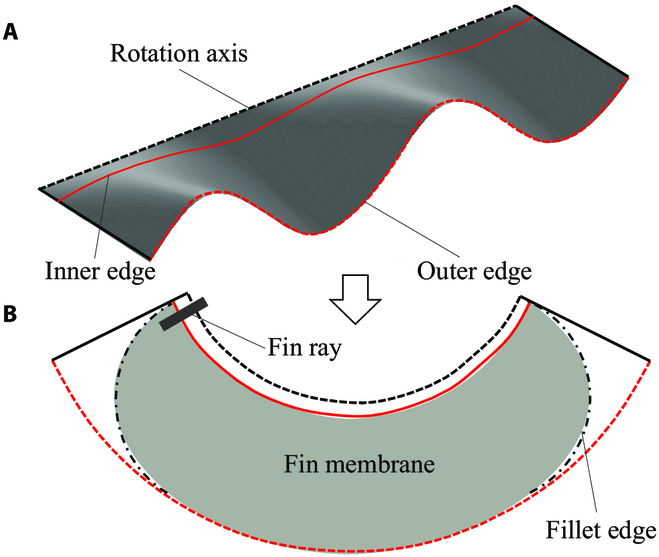
Fin membrane design process. (A) Simulated wave pattern. (B) Fin membrane sketch.

We design the shape of the flexible undulatory fins based on a certain wave pattern (*θ* = 30^∘^, *λ* = 290 mm, *l* = 580 mm, *h* = 225 mm). As shown in Fig. 5, the three-dimensional wave pattern can be approximated to a ring sector so that the fin membrane can be easily cut from a plane material. After testing different materials and thickness of the fin membrane on a force measurement platform [12], 4-mm silicone sheet is chosen as the fin membrane material.

**Fig. 6.**
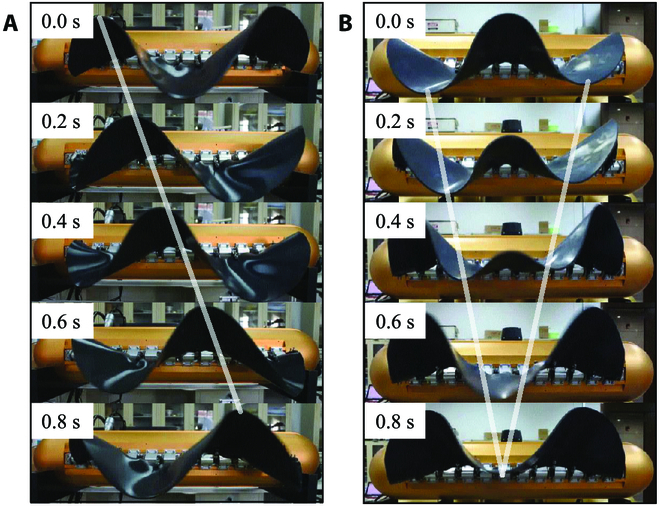
Snapshot sequences of the fins while undulating. (A) With parameters of *f* = 1 Hz, *η* = 0. (B) With parameters of *f* = 1 Hz, *η* = 0.5. The translucence lines connect peaks or troughs to better show the wave directions.

To define the proportion of the counterpropagating waves, a position ratio *η* = *d*/*l* is designed. When *η* equals 0 or 1, the pattern of counterpropagating waves can also be considered a normal sinusoidal wave pattern. Therefore, *f* and *η* are used as the control parameters of the undulatory fins. Figure 6 shows how the real flexible fins are undulating under certain parameters.

#### Hydrodynamic analysis of the undulatory fins

**Fig. 7.**
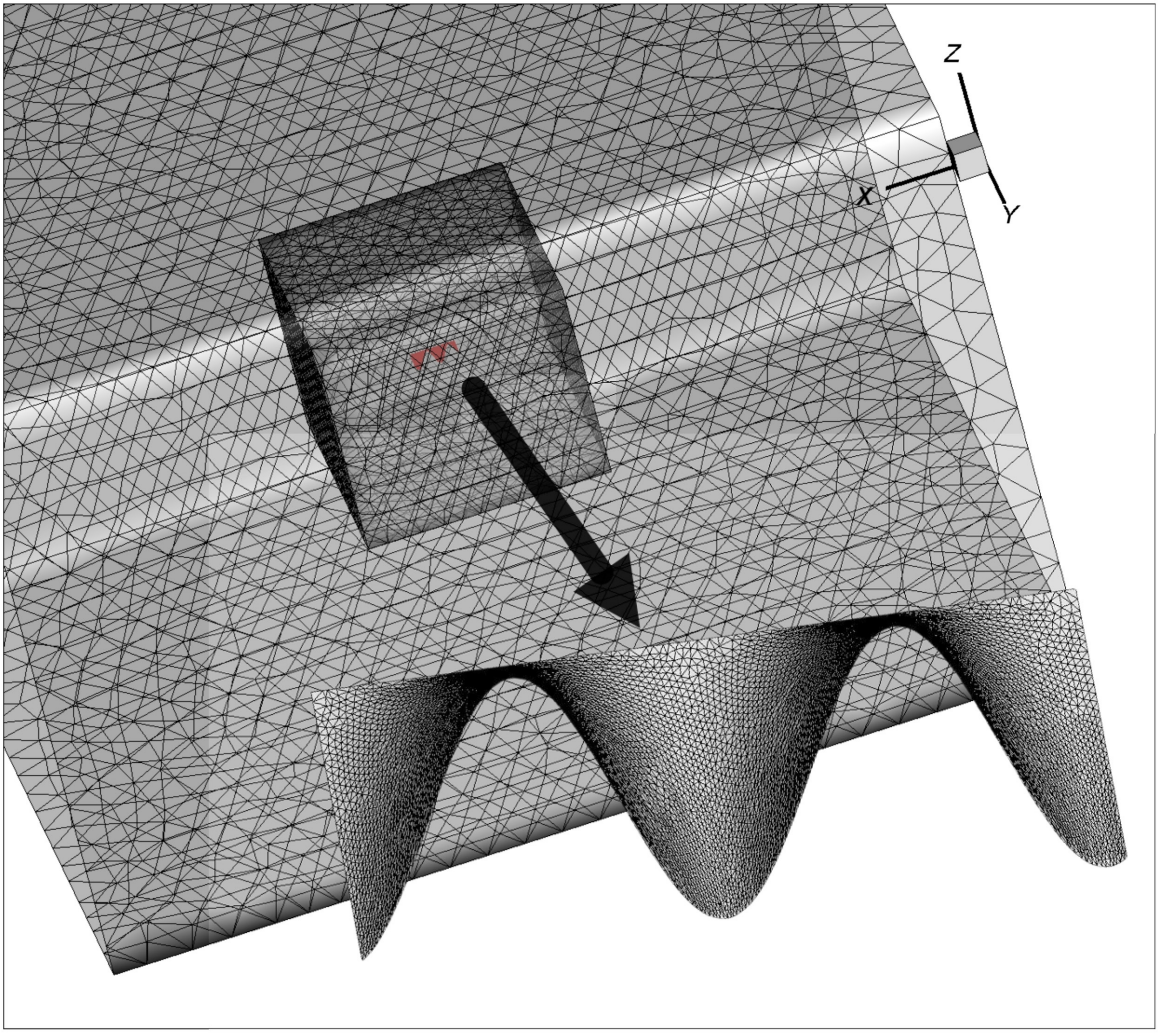
The mesh design in Fluent for undulatory fins and the flow field.

The design of a kinematic mechanism needs to be tested for its propulsive force. In order to confirm the reasonability and efficacy of the undulatory fins, we use computational fluid dynamics (CFD) technology to examine the hydrodynamic mechanism. The structural model of the flexible undulatory fins, the flow field model, the mesh model, and the dynamic mesh motion rules are all built using ANSYS series software to simulate the hydrodynamic situation of the undulatory fins under various motion parameters. The structural model of the undulatory fins is created in the ANSYS ICEM program at a 1:1 scale, as shown in Fig. 7. A local encrypted flow field is created around the undulatory fins, and a turbulence model is employed to improve simulation accuracy because the undulatory fins produce complex flow motion when they are in motion.

**Fig. 8.**
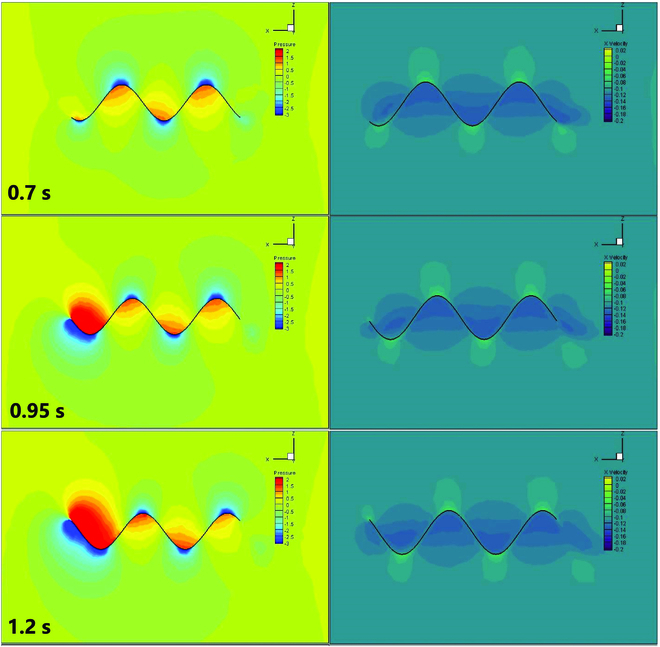
The contour of one undulatory fin surface pressure field and velocity field based on CFD at various times.

**Fig. 9.**
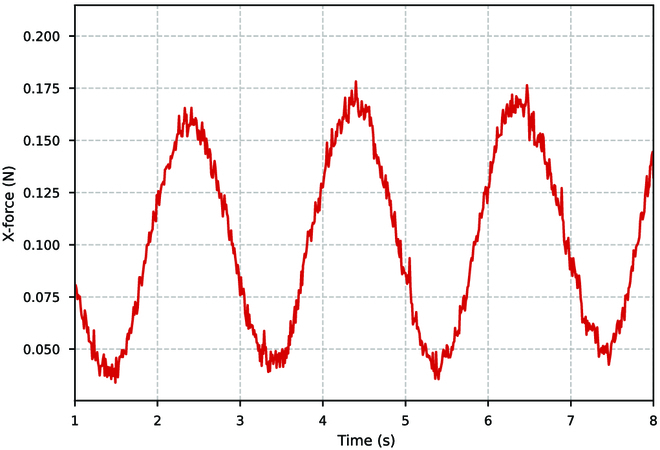
Force generated by the undulatory fins in the *x*-axis forward direction.

At a water velocity of 0.1 m/s, we adjust the undulatory fins’ vibration frequency to 0.5 Hz. After the water flow has stabilized, we estimate the pressure and velocity distribution around the undulatory fins’ surface. Due to the fins’ fluctuating motion, the surface produces pressure differences as illustrated in Fig. 8. The pressure difference on the surface of the undulatory fins generates the driving force for the mechanism. In the velocity contour, it can also be seen that the undulatory fins, when undergoing sinusoidal wave fluctuations, push the water backward, making the velocity of the water flow around the undulatory fins greater than 0.1 m/s. This results in a forward reaction force as a driving force. To obtain the variation of the driving force in the *x*-axis direction of the entire undulatory fins, as illustrated in Fig. 9, we calculate the overall pressure using Tecplot. The plot of the variation of the propulsive force shows that the dynamics of the undulatory fins take the form of a sinusoidal function, which is also periodic in nature. This paper only shows the generated thrust by the undulatory fins at 0.5 Hz, and as the frequency of fluctuation of the undulatory fins increases, the generated thrust increases accordingly. If the different waveforms are changed, the generated thrust will also have different characteristics in different directions. For example, a collision wave will produce a distinct upward and downward driving force.

### System Description

This section presents a description to our BUV and the control task. First, the BUV and its mathematical model are introduced (see the “BUV introduction” section). Then, the discontinuous path following task is illustrated with certain objectives (see the “Control task illustration” section).

#### BUV introduction

**Fig. 10.**
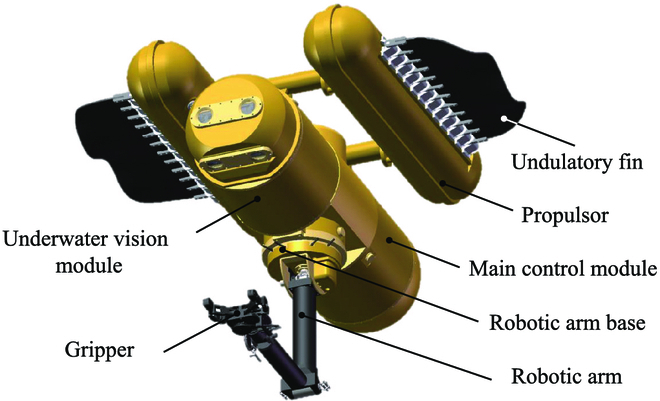
Schematic structure of the BUV with two sets of the flexible undulatory fins.

The mechanical design of the BUV is illustrated in Fig. 10. It consists of many modular-designed compartments: two sets of the flexible undulatory fins, a 5-degree-of-freedom robotic arm, a vision module, and a control module. Related parameters of the BUV are listed in Table.

**Fig. 11.**
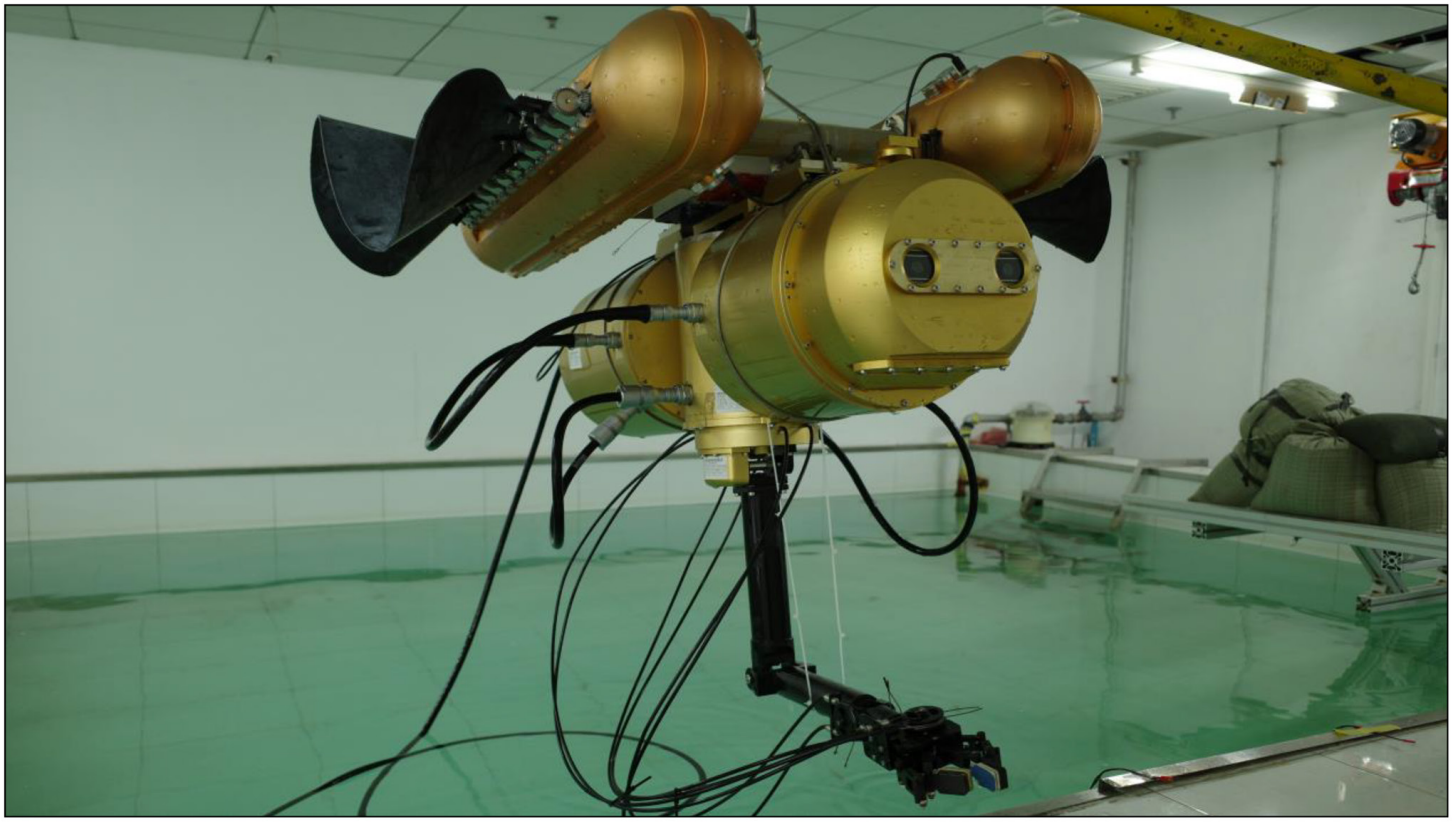
Entity model of the BUV.

**Table.** Parameters of the BUV

Width	Length	Height	Mass	Buoyancy
1,210 mm	1,200 mm	950 mm	120.6 kg	1,183.20 N

The control tasks in this paper are conducted on a planar underwater space (Fig. 11). Therefore, only two two-dimensional coordinates are considered. In the world coordinate system *E-xy*, the position and orientation of the BUV can be described as ***χ*** = [*x*, *y*, *ψ*]^T^. In the inertial coordinate system *O-uv*, the velocities of the BUV are ***ν*** = [*u*, *v*, *r*]^T^, and the resultant force and moment on the BUV are ***τ*** = [*X*, *Y*, *N*]^T^. Then, kinematics and dynamics of the BUV can be represented as:

$$\overset{\cdot }{\chi }=J\left(\psi \right)\nu ,$$ (1)

and

$$M\overset{\cdot }{\nu }=-C\left(\nu \right)\nu +\tau_{p}\left(a\right)-\tau_{h}\left(\nu ,\overset{\cdot }{\nu }\right)-\tau_{d},$$ (2)

where $J\left(\psi \right)\in \mathrm{SO}\left(3\right)$ is the coordinate transformation matrix (from *O-uv* to *E-xy*). $M\in {\mathbb{R}}^{3\times 3}$ is the generalized mass matrix. $C\left(\nu \right)\in {\mathbb{R}}^{3\times 3}$ is the matrix of Coriolis and centripetal force.$\tau_{p}\left(a\right)\in {\mathbb{R}}^{3}$ describes the force and moment generated by two sets of the undulatory fins. They are determined by the control parameters of fins $a={\left[\;f_{l},\eta_{l},f_{r},\eta_{r},\right]}^{T}$, where *l* and *r* indicate left and right fins, respectively. $\tau_{h}\left(\nu ,\overset{\cdot }{\nu }\right)\in {\mathbb{R}}^{3}$ describes hydrodynamic effect. ***τ****_d_* $\in {\mathbb{R}}^{3}$ denotes other disturbances.

#### Control task illustration

**Fig. 12.**
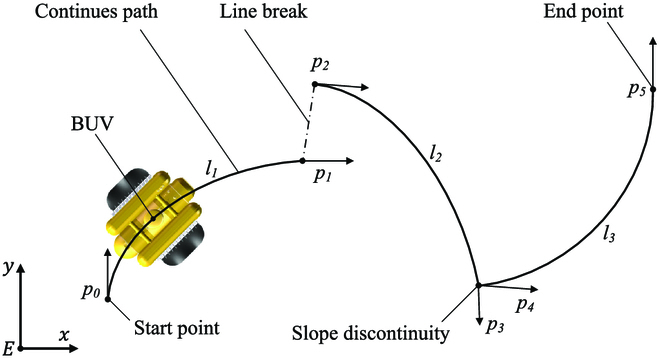
Illustration of the discontinuous path following task. *p_i_*(*i* = 1, 2, …, 5) are vectors, which indicate positions and slops of line ends.

The discontinuous path following task is illustrated in Fig. 12. The BUV starts at $p_{0}$ and then follows paths $l_{1}$, $l_{2}$, and $l_{3}$ in turns to reach the end point $p_{5}$. The description of discontinuous line is displayed in two aspects: first, the literally break of the line, which is illustrated by the gap between $p_{1}$ and $p_{2}$, and, second, the discontinuity of slops, which is shown by the slope variation from $p_{3}$ to $p_{4}$. The BUV should conquer these discontinuous line situations and successfully follow all the continuous paths in turns. To be more specific, the BUV should accomplish these objectives to achieve the discontinuous path following task:

1. In every continuous path, the BUV should follow the path while keeping its geometric center all the way along the path (e.g., $l_{1}$).

2. When encountering a discontinuous path situation, the BUV should switch path by changing its position and attitude from the end of the last path to the start of the next path (e.g., from $p_{1}$ to $p_{2}$). The BUV should also stop and keep itself stable at the end point (e.g., $p_{5}$).

### Control Scheme

**Fig. 13.**
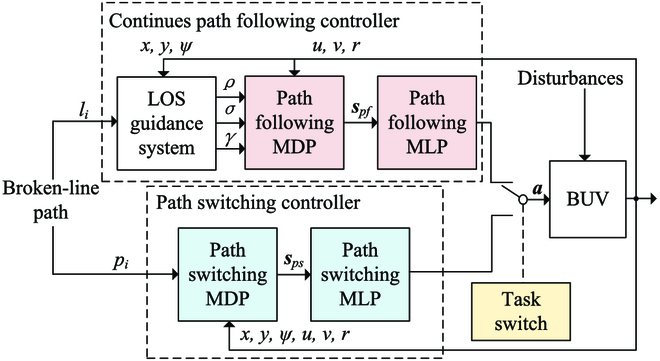
Block diagram of the discontinuous path following controller.

The discontinuous path following controller is mainly composed of three parts, as shown in Fig. 13. Two DRL-based subcontrollers are designed to separately face the continuous path following task (objective 1 in the “Control task illustration” section) and path switching task (objective 2 in the “Control task illustration” section). A task switch is designed to observe the environment and decides which subcontrollers should be applied toward different situations. In this section, first, the MDPs of path following task and path switching task are introduced (see the “Markov decision processes modeling” section). Second, control multilayer perceptrons (MLPs) for the MDPs are designed, which can transform state into action to directly control the BUV (see the “Multilayer perceptrons designing” section). Third, DRL methods are used to train the two MLPs, and related algorithm and the task switch are introduced (see the “Algorithms” section).

#### MDP modeling

A standard MDP consists of four parts: an action space A, a state space S, a one-step reward function r:S×A→ℝ, and a one-step transition probability $p\left(s_{t}|s_{t-1},a_{t-1}\right)$. They are designed as follows:

1. The action a∈A. The action defines how the BUV interacts with the environment. It is designed as:

$$a={\left[\;f_{l},\eta_{l},f_{r},\eta_{r},\right]}^{T},$$ (3)

which also indicates the control parameters of the undulatory fins in the “BUV introduction” section. This a is used in both path following and path switching tasks so that the control signals can be seamlessly sent to the BUV when the subcontroller changes.

**Fig. 14.**
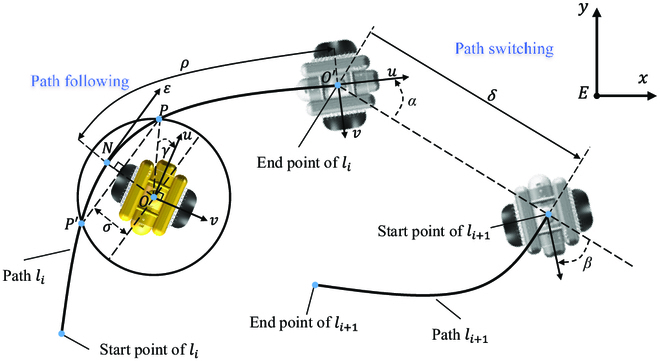
Illustrations of the LOS guidance system for the path following task and the path switching task.

2. The state s∈S. The state describes how the BUV observes the environment, and it is different in the two subcontrollers. As for path following, a guidance system is required to observe the paths and summary necessary information to guide the BUV. Therefore, a line-of-sight (LOS) guidance system [21] is designed as shown in Fig. 14. *ρ*, *σ*, *γ* can be obtained from the guidance system, where *ρ* is the remaining path length from the start point to the end point, which can also be called along-track error. *σ* is the cross-track error, and *γ* shows the attitude of the BUV. Combined with the velocities of the BUV, the state for path following MDP is designed as:

$$s_{\mathrm{pf}}={\left[\rho ,\sigma ,\gamma ,u,v,r\right]}^{T},$$ (4)

Similarly, when encountering the discontinuous line situation, the BUV needs to switch its position from the end of path $l_{i}$ to the start of the path $l_{i+1}$ as shown in Fig. 14, where *α* is relative attitude of the BUV, *β* is the relative attitude of the target pose, and *δ* is the distance from the BUV to the target pose. The state for path switching MDP is designed as:

$$s_{\mathrm{ps}}={\left[\alpha ,\beta ,\delta ,u,v,r\right]}^{T}.$$ (5)

3. The reward function $r\left(s\right)$. The reward function guides the BUV to achieve the tasks, and it is also separately designed for each subtask. As for path following, the BUV should keep on tracking the path by simultaneously reducing *ρ*, *σ*, *γ*. So, the path following reward function is designed as:

$$r_{\mathrm{pf}}\left(s_{\mathrm{pf}}\right)=-k_{1}\rho^{2}-k_{2}\sigma^{2}-k_{3}\gamma^{2},$$ (6)

where $k_{i}\;\left(i=1,2,3\right)$ are weight coefficients. As for path switching, the BUV should reduce *δ* and ∣*α* − *β*∣ to reach the target pose, and minimize its speed to stop at the target pose. Therefore, the path switching reward function is designed as:

$$r_{\mathrm{ps}}\left(s_{\mathrm{ps}}\right)=-k_{4}\delta^{2}-k_{5}{\left(\alpha -\beta \right)}^{2}-k_{6}\nu^{T}M\nu ,$$ (7)

where $k_{i},\left(i=4,5,6\right.$ are also weight coefficients. $\nu^{T}M\nu$ describes the kinetic energy of the BUV. The selection of $k_{1}$ to $k_{6}$ is a multi-objective optimization problem. Different parameter sizes represent varying significance on control goals. The optimal performance parameters for training are finally obtained through constant parameter adjustment in actual engineering.

#### MLP designing

**Fig. 15.**
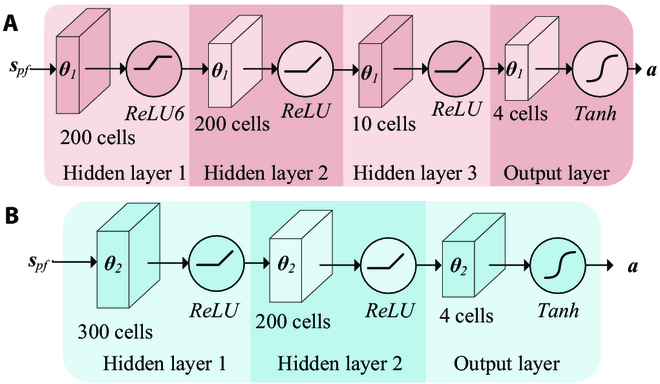
Block diagram of MLPs. (A) MLP of path following. (B) MLP of path switching.

In this study, we employ MLPs, a type of neural network, to design the controllers for our control task. MLP utilizes a nonlinear mapping to directly transform inputs into outputs. Our aim is to develop end-to-end controllers for our BUV, and the mapping process is approximated using MLP. The MLPs of two subcontrollers are designed as shown in Fig. 15. The MLP for path tracking contains 1 input layer, 3 hidden layers, and 1 output layer. Neurons in the input layer receive state information from the BUV. The network structure of the hidden layer is 200 × 200 × 10. The MLP for path switching contains 1 input layer, 2 hidden layers, and 1 output layer. The network structure of the hidden layer is 300 × 200. The neurons in the output layer of both MLPs output the motion parameters of the BUV, using the Tanh activation function.

#### Algorithms

**Fig. 16.**
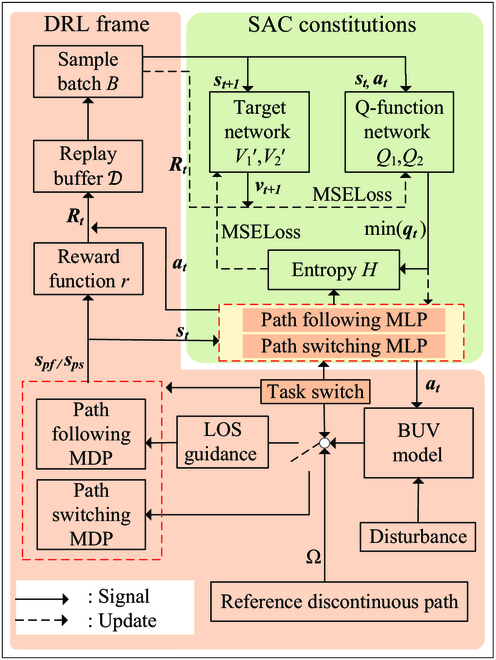
Schematic of the DRL algorithm.

To obtain the correct weights *θ* for MLPs, we employ the soft actor-critic (SAC) [22] DRL framework for training. The schematic of the DRL training algorithm is shown as Fig. 16. We build the simulation environment with added disturbances based on the dynamics model of BUV and use the task switching mechanism to select the MLP for training and the acquisition of the state at each moment. Then, we utilize the designed reward function to evaluate the current state and add them into the replay buffer for the SAC algorithm’s sampling training. Through the design of different MDPs and reward functions, we train two subcontrollers: the path following controller and the path switching controller. In practical use, input parameters of the DRL algorithm are chosen as number of reinforcement learning episodes *M* = 15000, number of control steps per episode *N* = 500, capacity of the replay buffer *R* = 20000, batch size *n* = 64, reward discount *γ* = 0.9, trade-off coefficient *δ* = 0.001, and polyak *ϵ* = 0.001.

## Conclusions

In this paper, we validate the effectiveness of the control scheme of DRL for the BUV equipped with two sets of the flexible undulatory fins. The control scheme harnesses the strong coupling and nonlinear thrust characteristics among the undulatory fins. By training the model, we obtain the path following controller as well as the path switching controller. The DRL control method enables the BUV to efficiently navigate through complex underwater environments. The results confirm the controllers’ capability to accurately track predefined trajectories and smoothly switch between different paths.

Future research will concentrate on other learning-based control schemes on our BUV toward tasks like obstacle avoidance control based on multimodal sensing.

## Data Availability

The data that support the findings of this study are available from Institute of Automation, Chinese Academy of Sciences. Restrictions apply to the availability of these data, which were used under license for this study. Data access inquiries can be sent to chuhohngfei2022@ia.ac.cn.
